# Metabolic trajectories of diabetic ketoacidosis onset described by breath analysis

**DOI:** 10.3389/fendo.2024.1360989

**Published:** 2024-05-01

**Authors:** Mo Awchi, Kapil Dev Singh, Sara Bachmann Brenner, Marie-Anne Burckhardt, Melanie Hess, Jiafa Zeng, Alexandre N. Datta, Urs Frey, Urs Zumsteg, Gabor Szinnai, Pablo Sinues

**Affiliations:** ^1^ University Children’s Hospital Basel, Basel, Switzerland; ^2^ Department of Biomedical Engineering, University of Basel, Basel, Switzerland; ^3^ Department of Clinical Research, University of Basel, Basel, Switzerland

**Keywords:** diabetic ketoacidosis, mass spectrometry, breath analysis, metabolomics, ICU

## Abstract

**Purpose:**

This feasibility study aimed to investigate the use of exhaled breath analysis to capture and quantify relative changes of metabolites during resolution of acute diabetic ketoacidosis under insulin and rehydration therapy.

**Methods:**

Breath analysis was conducted on 30 patients of which 5 with DKA. They inflated Nalophan bags, and their metabolic content was subsequently interrogated by secondary electrospray ionization high-resolution mass spectrometry (SESI-HRMS).

**Results:**

SESI-HRMS analysis showed that acetone, pyruvate, and acetoacetate, which are well known to be altered in DKA, were readily detectable in breath of participants with DKA. In addition, a total of 665 mass spectral features were found to significantly correlate with base excess and prompt metabolic trajectories toward an in-control state as they progress toward homeostasis.

**Conclusion:**

This study provides proof-of-principle for using exhaled breath analysis in a real ICU setting for DKA monitoring. This non-invasive new technology provides new insights and a more comprehensive overview of the effect of insulin and rehydration during DKA treatment.

## Introduction

Breath analysis, encompassing breathomics or breath metabolomics, is an emerging field that offers a non-invasive and convenient approach to investigate the metabolic processes occurring within the human body. It involves the collection and analysis of volatile organic compounds (VOCs) present in exhaled breath, which are reflective of various biochemical reactions and metabolic pathways taking place in different organs and systems (1). A prominent example known for decades (2) is acetone, whose concentration in exhaled breath is influenced by adipocyte fatty acid release and hepatocyte uptake as well as liver production of ketone bodies subsequent to excessive fatty acid catabolism during nutrient deprivation (2, 3). Advancements in analytical techniques, such as mass spectrometry and ionization techniques, have greatly facilitated the identification and quantification of these VOCs, enabling researchers to better understand the complex metabolic signatures associated with different physiological and pathological conditions (4, 5). In our study, we employed a breath analysis technique known as secondary electrospray ionization high-resolution mass spectrometry (SESI-HRMS), a technique with detection limits in the sub-ppt range (6). SESI-HRMS is a powerful analytical tool that combines secondary electrospray ionization with high-resolution mass spectrometry, allowing for the sensitive and comprehensive detection of a wide range of VOCs in breath samples (7). In the field of endocrinology, breath analysis holds great promise as a tool for investigating the metabolic alterations that occur in various endocrine disorders, including diabetes (8, 9). As metabolism plays a central role in endocrine function, alterations in metabolic pathways can significantly impact hormone regulation and overall endocrine homeostasis. Therefore, the ability to non-invasively monitor these metabolic changes through breath analysis, specifically utilizing the SESI-HRMS technique, provides a unique opportunity to gain insights into the underlying pathophysiology of endocrine disorders. Building upon this foundation, the present study focuses on the application of SESI-HRMS-based breath analysis in the context of diabetic ketoacidosis (DKA), a serious complication of diabetes characterized by severe insulin deficiency and the accumulation of ketone bodies. DKA requires prompt medical intervention, including insulin administration and rehydration therapy, to restore metabolic balance and prevent further complications (10, 11). Although DKA has been extensively studied, the exact metabolic mechanisms leading to its onset and progression remains incompletely understood (12). By leveraging the power of SESI-HRMS-based breath analysis, we aim to explore novel insights into the metabolic changes occurring in DKA patients during recovery from DKA, the transition from a catabolic state to an anabolic state following the initiation of insulin and rehydration therapy. DKA is a consequence of insulin deficiency characterized by hyperglycemia, acidosis and ketonemia. Hyperglycemia and ketogenesis are caused by insulin deficiency and secondary increase in counterregulatory hormones such as cortisol, catecholamines and glucagon. Decreased peripheral glucose utilization, increased proteolysis and glycogenolysis contribute to hyperglycemia (10, 11). Increased lipolysis results in increased free fatty acid levels and consecutively in hepatic ketone production. Ketones are acidic and cause metabolic acidosis. In clinical practice, urine ketone measurements allow semiquantitative measurement of acetoacetate and acetone, while recently available bed-side blood measurements of beta-hydroxybutyrate allow precise quantification and have a high accuracy for prediction of DKA (10). As recently shown, plasma beta-hydroxybutyrate measurements also allow to precisely define resolution of DKA independently of pH or bicarbonate (13).

The underlying hypothesis of our pilot study is that SESI-HRMS-based exhaled breath analysis can provide valuable metabolic information that captures the dynamic shifts in metabolic pathways during resolution of DKA. By monitoring changes in VOC profiles over time, we anticipate uncovering novel metabolic signatures associated with insulin and rehydration therapy of DKA. This report presents the design and preliminary findings of our study, which employed the SESI-HRMS technique to analyze breath samples collected from a cohort of DKA patients. Our approach integrates clinical and biochemical data, enabling us to correlate the observed breath profiles obtained through SESI-HRMS with key clinical indicators of DKA severity and treatment response. By elucidating the metabolic dynamics of DKA patients through SESI-HRMS-based breath analysis, we aim to contribute to a deeper understanding of the pathophysiology of DKA treatment and recovery (14).

## Materials and methods

This observational, longitudinal study was conducted at the University Children’s Hospital Basel, Basel, Switzerland. The Ethics Committee of North–Western and Central Switzerland ID 2020-00778 (EBECA) approved the study and written informed consent was obtained. The study has been registered in https://clinicaltrials.gov/ct2/show/NCT04461821.

This study included participants aged 5-20 years old with and without type 1 diabetes (T1D). Participants with T1D were categorized according to glycemia and pH as i) with DKA (pH<7.3, base excess (BE) <-2, blood glucose>11 mmol/L, and ketonemia); ii) with hyperglycemia (pH>7.3, BE <-2, blood glucose>11 mmol/L), and ketonemia, or iii) euglycemic. DKA participants admitted to the intensive care unit (ICU) were treated according to international guidelines (10).

Participants with epilepsy were treated with antiseizure medication in form of a mono- or polytherapy and represented the control group without T1D. Patients under ketogenic diet or modified Atkins diet were excluded.

Breath was analyzed using custom-made Nalophan bags inflated with 2L of breath (15), and subsequently infused into the mass spectrometer within 15 minutes of collection. The mass spectrometer operated using an interface which consisted of a SESI source (FIT, Spain) coupled to an HRMS instrument (Orbitrap, Thermo Fisher, USA). The instrumental setting for the mass spectrometer were as follows: sheath gas flow rate of 60, auxiliary gas flow rate of 2, spray voltage at 2.8 kV in positive ion mode, the capillary temperature of 275°C, and S-lens RF level of 55. A nano-spray was created by utilizing 0.1% formic acid in water (Sigma Aldrich), whereby the current ranged between 70 and 140 nA. SESI sampling line temperature was set at 130°C and the ion source at 90°C. The MS was operated in full-scan mode ranging from 55-825 m/z, with a maximum inject time of 500 ms, and automatic gain control target of 10^6^. The mass spectral resolution was set at 140,000 at m/z 200. The mass spectrometer was calibrated weekly and a suitability test [see (16) for details] was performed before every day of measurements.

Raw centroid and profile mass spectra were accessed using in-house C# console apps based on Thermo Fisher Scientific’s RawFileReader (version 5.0.0.38). Centroid and profile mass spectra were recalibrated using reference peaks. Zeros were imputed using the regression on order statistics method (17). This procedure resulted in a 107 x 3505 data matrix. The time points between the clinical measurement and breath measurement differed variably, to obtain a BE value during the breath measurements, BE values were interpolated using linear interpolation on minute resolution for the whole-time frame (i.e. during their ICU stay). Time points overlapping with breath measurements were extracted and the corresponding BE value was used.

## Data and resource availability

The data set generated during and analyzed in the study is available upon reasonable request from the corresponding authors.

## Results

**Figure 1:**
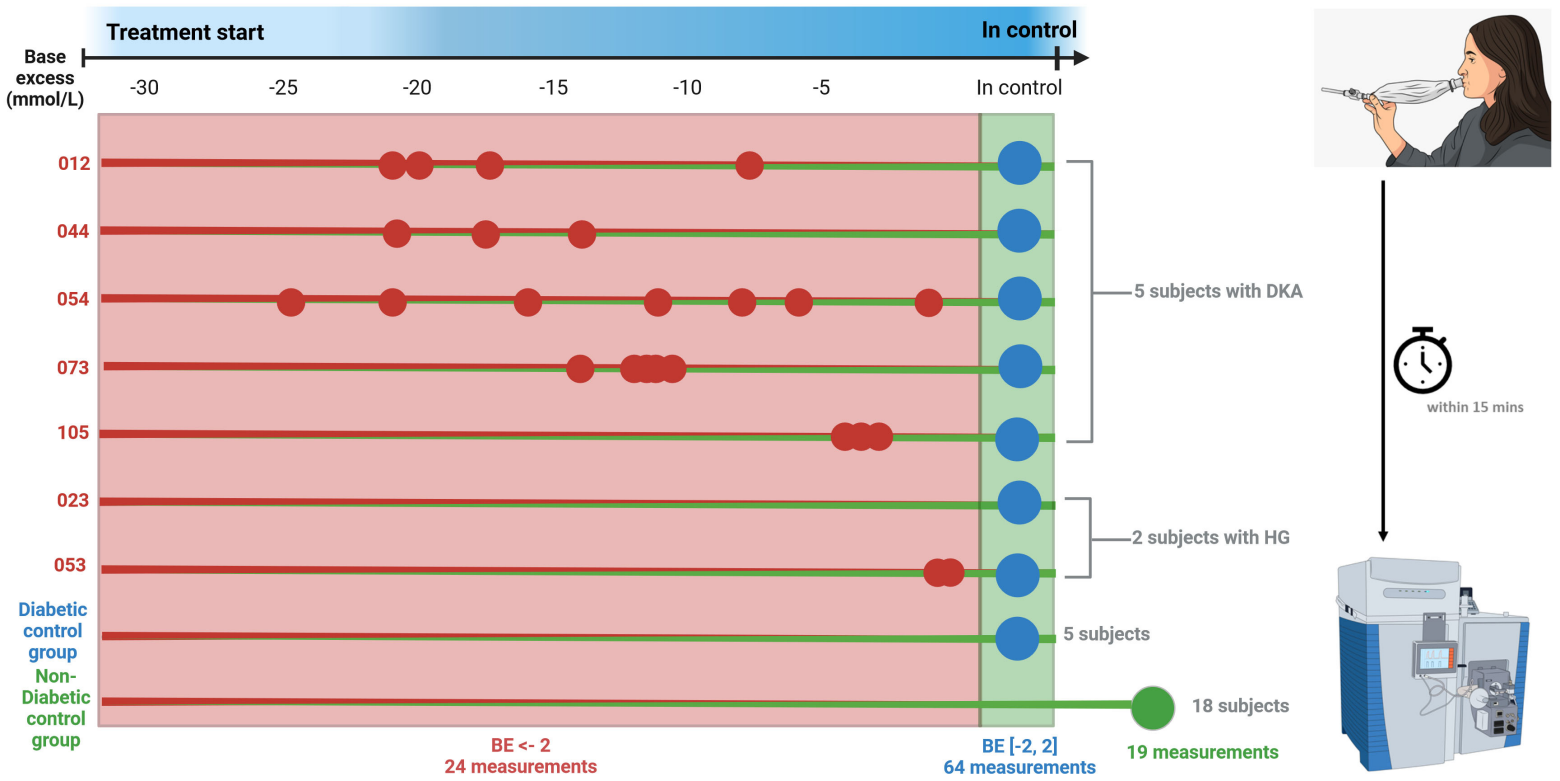
Overview of patient measurements. Small dot indicates single breath measurement, big dot indicates multiple breath measurements. Red numbers indicate DKA, blue in control and green non-diabetic. The breath samples were collected and measured within 15 minutes after collection. The total measurements were the base excess was below -2 totaled 24, whereas 64 measurements were made when diabetic participants were in control and 19 measurements of non-diabetic control measurements. Image was generated with the use of BioRender.

We recruited 30 participants (mean age 11.9 years, range 5.2-19.7 years, 60% male), five of which were DKA participants admitted to the ICU. The biochemical characteristics of the DKA participants while treated at the ICU are shown in **Supplementary Tables S1-S5**. Longitudinal breath measurements (n = 32) of these 5 DKA participants were recorded during DKA and after full recovery from DKA. Similarly, two participants with hyperglycemia (HG) were monitored during hospitalization, providing a total of n = 6 breath measurements (**Supplementary Tables S6**, **S7**). In total, these 7 participants contributed n = 24 breath measurements while their BE was below -2 mmol/L and n = 14 with while their BE was stabilized in the range -2 to +2 mmol/L. In addition, 12 euglycemic patients (i.e., [-2 < BE < 2] mmol/L), including revisits from DKA and HG participants, provided a total of n = 50 measurements. Finally, 18 participants without T1D treated for epilepsy contributed n = 19 breath measurements. This resulted in a total of N = 107 individual breath measurements (**Figure 1**, **Supplementary Figure S1**).

**Figure 2:**
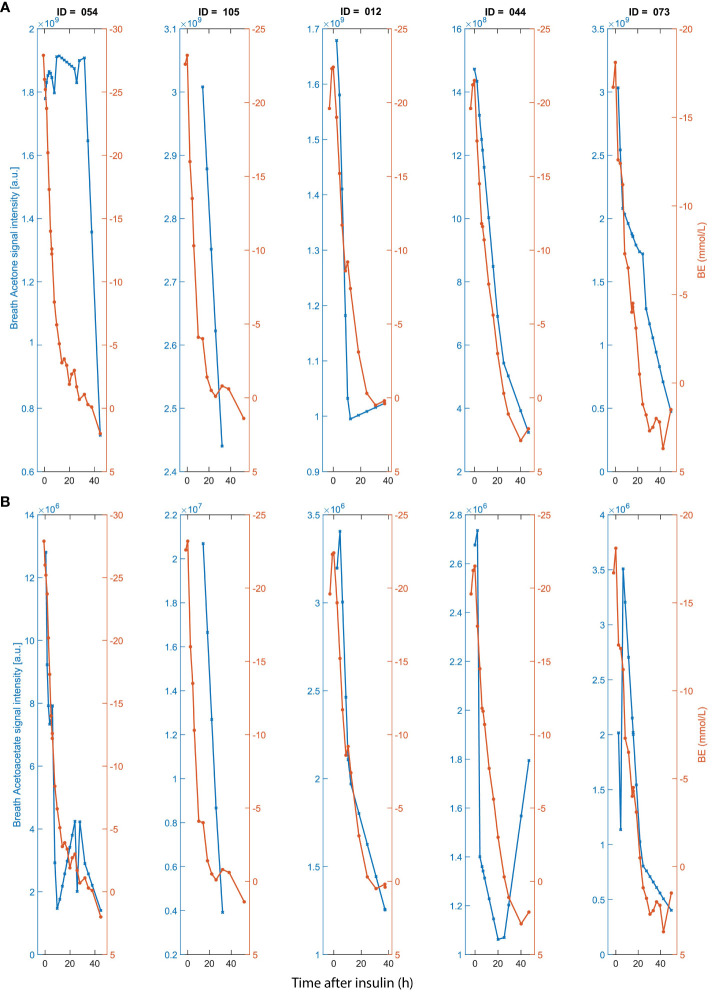
Time traces of **(A)** breath acetone (left y-axis); **(B)** and acetoacetate (left y-axis); in comparison with BE (right y-axis in a and b) for five DKA participants admitted to ICU after start of insulin administration and rehydration therapy. X-axis represents time after start of iv insulin. Breath timepoints were linearly interpolated to match with the clinical time-points.

In the first part of the study, we assessed the utility of breath analysis for monitoring the normalization of DKA after start of intravenous (iv) insulin and rehydration therapy. Breath acetone and breath acetoacetate follow a pattern mirroring BE (**Figure 2**). Additionally, we observed a significant correlation (r = 0.51; p ~ 2.82x10^-8^) between breath acetone and AcAc (**Supplementary Figure S2**), which is consistent with their known biochemical interplay.

**Figure 3:**
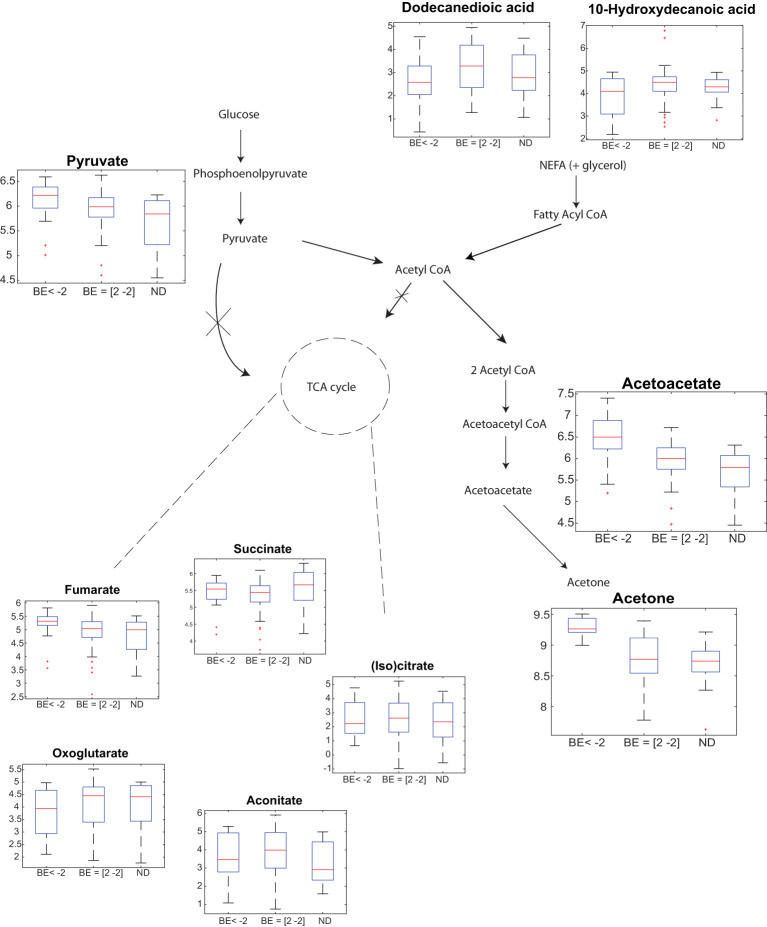
Breath analysis captures generally accepted DKA-altered pathways. Participants with T1D with BE < -2 show a significant partial up-regulation of pyruvate, and significant upregulation acetone and acetoacetate vs participants with T1D with normal BE values (i.e. +/- 2) and participants without T1D. In contrast, exhaled free fatty acids are downregulated. Y-axis in the boxplots represent: Log10(signal intensity) [a.u.]. Figure adapted from Wallace et al. (18).

For this pilot study, we aimed to elucidate the generally accepted pathways, previously reported by Wallace et al. (18). As shown in **Figure 3** (please note Log10 scale for all metabolites), we observe downregulated levels of non-esterified fatty acids (NEFAs) for diabetic patients with BE < -2 vs BE -2/+2 (p < 0.05, **Supplementary Table S8**). This data corroborates the hypothesis positing that the presence of NEFAs as substrates is crucial for the biosynthesis of ketone bodies (19). Further, it has been shown by Mingrone et al. (20) that dodecanedoic acid might represent a fuel substrate immediately available for tissue energy requirements eventually explaining rather decreased than elevated levels during DKA. However, this observation should be interpreted cautiously, given the moderate level of statistical significance and the fact that no significant differences were found between NEFAs (i.e. Dodecanedioic acid and 10-Hydroxydecanoic acid) levels between diabetic patients with BE < -2 and ND, nor between BE -2/+2 vs ND.

Overall, the increased lipolysis results in formation of ketone bodies. As expected, significant upregulation is observed for acetone and AcAc (**Figure 3**). Pyruvate also shows an upregulated trend. The TCA cycle however, did not show significant alterations (with the exception of fumarate). **Supplementary Table S8** lists the multiple comparison statistical values for all these DKA-relevant exhaled metabolites, including all previously identified fatty acids in breath (21, 22).

**Figure 4:**
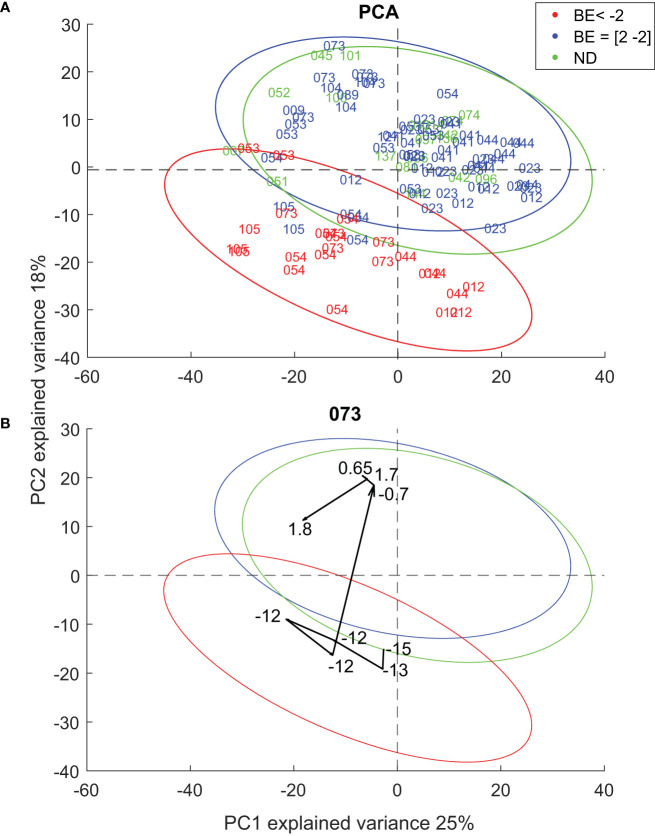
**(A)** PCA plot of all patients **(B)** Participant 073 shows trajectory towards in-control state as it progresses towards homeostasis.

Once we confirmed the soundness of the proposed breath analysis approach by confirming that AcAc and acetone mirror BE (**Figure 2**), as well as an overall consistent picture of known altered metabolic pathways (**Figure 3**), we further explored to what extent the comprehensive exhaled metabolic fingerprint may provide novel metabolic insights into DKA. This was pursued by investigating the correlation between BE values and the rest of the metabolic features detected in breath. A total of 665 features were found to significantly correlate (p < 0.05; q < 0.18) with BE (**Supplementary Table S9**). 219 features showed significant positive correlation while 446 features showed a significant negative correlation. Two examples of such features showing a significant positive and negative correlation with BE levels are shown in **Supplementary Figure S3**. The feature with a positive correlation (r = 0.67; q = 4x10^-3^) corresponds to a metabolite with molecular formula of C_7_H_14_O, while the one with a negative correlation with BE (r = -0.66; q = 5x10^-3^) could be mapped to C_8_H_6_O_3_. To further visualize the overall contribution of the exhaled metabolites to the associated DKA stage, we subjected the dataset to principal component analysis (PCA). **Figure 4A** shows the score plot of the first two PCs, explaining 43% of the variance. **Figure 4A** shows two distinct main clusters: participants with BE < -2 (lower quadrant; red data points) and the rest. Participants with and without T1D in euglycemia cluster together (i.e., overlapping 95% confidence interval (CI) ellipses). This indicates that, even though the participants are highly heterogeneous (i.e., participants with epilepsy and T1D in euglycemia), they are equally homeostatic from a metabolic acidosis point of view, and their breath fingerprints capture such a state.

**Figure 4B** shows how this technique can be used to map the individual metabolic trajectories of DKA participants after the start of iv insulin and rehydration treatment. It clearly shows how the trajectory migrates from the lower half of the PCA space (catabolic state) when the BE values ranged between -15 and -12, to the upper half (anabolic state) when the BE value was -0.7. The remaining DKA patients’ trajectories are shown in **Supplementary Figures S4-S7**. For patient 012, all measurements were recorded while BE was well below -2, and consistently his/her breath metabolic trajectory remained within the -2 < BE 95% CI ellipse. Patient 044 showed a similar behavior as 073, with a clear scattering around the lower half of the space while BE was well below -2, to then sharply transition to the in-control region once the BE was stabilized to -1.4. In contrast, patients 054 and 105 showed that they approached the in-control region as their BE stabilized, but they hardly reached the transition boundary between both regions. Somehow, these two patients seem to show a less “elastic” metabolic recovery. This is supported by the gradient of ketone bodies (**Supplementary Figure S8**), which shows a lagging behavior for patients 054 and 105, whereby the global minimum of the first derivative of ketone bodies time profiles occurs 8h and 10 h after insulin administration, respectively. In contrast, for the other three patients the minimum occurs 4-6 h after insulin treatment. Interestingly, these two patients were known T1D patients with insufficient metabolic control and a history of precedent DKA, while the other three patients experienced DKA at diagnosis of T1D. However, the interpretation of such differences between these two patients and the rest must be taken cautiously given the very limited sample size of our cohort.

**Supplementary Figures S9**, **S10** show the metabolic trajectories of the two HG patients (053 and 023) for which BE values were available. Again, the metabolic picture falls in the in-control domain, however, rather in the interface between both regions, especially for the lowest BE data points of 053.

## Discussion

We confirmed that the generally accepted DKA-associated perturbed pathways could be captured by exhaled metabolomics. A more untargeted approach further revealed metabolic trajectories of DKA participants drifting apart during the course of their switch from catabolic to anabolic state, especially for those newly diagnosed. Taken all together, this pilot study suggests the exhaled profiles can track individualized metabolic trajectories mirroring the ketonic state.

In the late 1960s, Tassopoulos et al. (2) were already able to show that breath-acetone correlates with plasma-beta-hydroxybutyrate (BHB) and in larger extent the clinical state of patients undergoing DKA treatment. Further studies have confirmed this association (23). We used these observations as an anchor point for our current study and to draw further conclusions. The current study used BE, a surrogate marker for acid/base disturbances in blood, to compare with breath acetone. As expected, a similar trend is observed between sequentially measured breath acetone and BE during DKA treatment (**Figure 2A**).

Our study also investigated the relationship between BE and other ketone bodies, specifically acetoacetate (AcAc) and BHB. AcAc, produced during lipolysis and the breakdown of ketogenic amino acids, can be converted to acetone through decarboxylation. Our findings demonstrated for the first time a similar trend between BE and the breath signal intensity of AcAc (**Figure 2B**), including participant 054, for which acetone showed a lack of association at the beginning of the time series. This suggests that exhaled AcAc might be a more robust surrogate marker for BE in high BE ranges, however, around stabilization (BE =2) AcAc shows high variability. Acetone however, shows a more reflective behavior in low BE ranges and therefore might be a more fit for purpose in the lower BE ranges. In contrast, no obvious association was found between the mass spectral feature corresponding to BHB and BE.

Apart from acetone and AcAc, previous reports have shown the capability of breath analysis by SESI-HRMS to capture large areas of the human metabolome (16). A portion of the metabolome covered by this breath analysis technique plays a significant role in DKA. For example, butanoate metabolism (24), tricarboxylic acid (TCA) cycle (25), amino acid metabolism (26) and fatty acid metabolism (27). Insulin deficiency causes increased lipolysis, ketogenesis and metabolic acidosis. The downregulated non-esterified acids show in **Figure 3** are in agreement with previously reported observations (27). The increased lipolysis results in formation of ketone bodies (28, 29). As expected, significant upregulation is observed for acetone and AcAc (**Figure 2**). In addition, the upregulated trend from Pyruvate is also in agreement with previously reported observations (30). Previous reports on the alterations of TCA cycle metabolites (31) do show significant alterations, the available literature on TCA cycle in the context of DKA is quite scarce, however.

Worth of note are the participants without diabetes 051 and 007 in **Figure 4**, whose data points fall well within the BE < -2 space. Interestingly, these two patients were receiving anti-seizure medication Sultiame, which inhibits carbonic anhydrase, resulting potentially in metabolic acidosis (32), which may explain why they are mapped within the BE < -2 PCA region. This in addition sheds light on one of the limitations of the study. The study monitors breath makers in relation to BE, which is a marker for acidity and basicity in blood. As seen with participants 051 and 007, the study therefore cannot exclude influence of confounding factors that can cause acidity in blood such as the above mentioned Sultiame, or diarrhea and excessive aspirin use (33). Additionally, the data interpretation of BE in relation to breath metabolites depicts a causal relationship between insulin and rehydration therapy on exhaled metabolites; however, further investigation is needed to ascertain the precise mechanisms underlying this association and to determine any potential confounding factors. Finally, our breath metabolomics data suggests a “less elastic” behavior observed in the reoccurring DKA patients (**Supplementary Figures S6**, **S7**), which is consistent with previous reports suggesting metabolic alterations of long-term poor glycemic control patients versus good glycemic control patients (34).

However, our feasibility study has limitations that require an open discussion: i) The sample size is clearly limited. Thus, further independent validation studies will be required; ii) The identification of the majority of the metabolites reported here remains unknown, limiting the possibility of further interpreting our results from a mechanistic point of view. Further specialized measurements will be required to elucidate the structure of the most relevant metabolites (35). iii) As mentioned previously, the technique interfaced with the clinical parameters, focus primarily on the acidity and basicity of blood, and participants with for example diarrhea, ketogenic diets, respiratory or kidney failures should be accounted for in future studies. Despite these noted limitations, it is fair to conclude that the method could be seamlessly integrated in a real ICU setting. It only requires inflating a breath sampling bag and the subsequent read-out is provided immediately after deflating the sample into the mass spectrometer, providing an attractive new approach to conveniently interrogate the metabolome of patients in a clinical setting. This opens new possibilities to integrate this technique for the monitoring, not only participants with T1D during DKA, but of other patients with altered metabolism (36).

## Data availability statement

The raw data supporting the conclusions of this article will be made available by the authors, without undue reservation.

## Ethics statement

The studies involving humans were approved by the Ethics Committee of North–Western and Central Switzerland ID 2020-00778 (EBECA) and written informed consent was obtained. The study has been registered in https://clinicaltrials.gov/ct2/show/NCT04461821. The studies were conducted in accordance with the local legislation and institutional requirements. Written informed consent for participation in this study was provided by the participants’ legal guardians/next of kin.

## Author contributions

MA: Formal analysis, Investigation, Data curation, Software, Visualization, Writing – original draft, Writing – review & editing. KS: Conceptualization, Methodology, Visualization, Writing – original draft, Writing – review & editing, Data curation, Formal analysis, Investigation, Software. SB: Writing – original draft, Writing – review & editing. MB: Writing – original draft, Writing – review & editing. MH: Writing – original draft, Writing – review & editing. JZ: Investigation, Writing – original draft, Writing – review & editing. AD: Writing – original draft, Writing – review & editing. UF: Conceptualization, Writing – original draft, Writing – review & editing. UZ: Writing – original draft, Writing – review & editing. GS: Conceptualization, Methodology, Resources, Supervision, Writing – original draft, Writing – review & editing, Investigation. PS: Conceptualization, Funding acquisition, Methodology, Project administration, Resources, Software, Supervision, Visualization, Writing – original draft, Writing – review & editing.
