# Supplementary material for: Metabolic trajectories of diabetic ketoacidosis onset described by breath analysis
Source: Front Endocrinol (Lausanne). 2024 May 1;15:1360989. doi: 10.3389/fendo.2024.1360989 (PMC11094216; doi:10.3389/fendo.2024.1360989)
Supplement: Supplementary file 1 [file DataSheet_1.pdf]

Metabolic trajectories of diabetic ketoacidosis onset described by breath analysis

Mo Awchi[1,2], Kapil Dev Singh[1,2], Sara Bachmann Brenner[1,3], Marie-Anne Burckhardt[1,3], Melanie Hess[1,3], Jiafa Zeng[1,2], Alexandre N. Datta[1,3], Urs Frey[1,3], Urs Zumsteg[1], Gabor Szinnai[1,3*], Pablo Sinues[1,2,*]

1. University Children's Hospital Basel, Basel, Switzerland.

2. Department of Biomedical Engineering, University of Basel, Basel, Switzerland.

3. Department of Clinical Research, University of Basel, Basel, Switzerland

*Gabor Szinnai gabor.szinnai@ukbb.ch

*Pablo Sinues pablo.sinues@unibas.ch

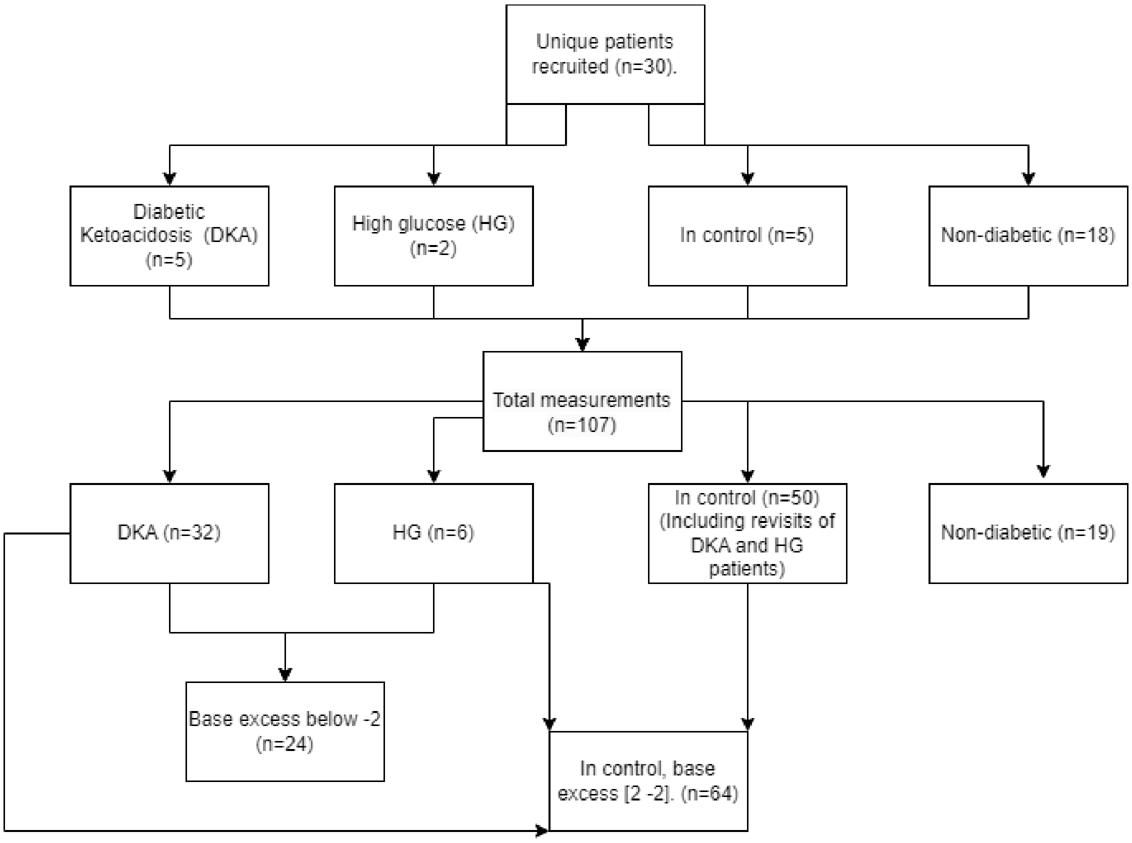

Figure S1 Study overview. Thirty unique patients were recruited of which five were diagnosed with diabetic ketoacidosis, two with high glucose, five were in-control and 18 were non-diabetic. All patients contributed to a total of 107 measurements. Twenty-four came from the DKA patients, eight from HG patients, 56 measurements came from patients who were in control (these included patients who were initially recruited during DKA state but were re-measured later when they were in control). The eighteen non-diabetic patients resulted in 19 measurements. After patient stratification, the measurements were divided in BE below -2, which totaled twenty-four measurements, in control (BE between 2 and -2) with 64 measurements.

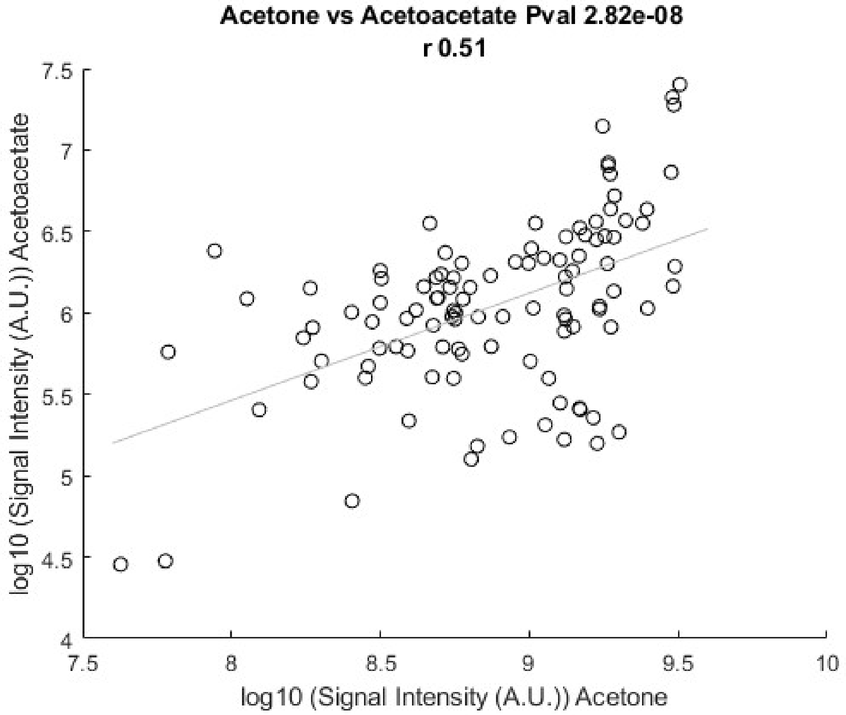

Figure S2 Acetone and acetoacetate show a significant correlation ($r = 0.51$; $p < 3e\text{-}8$) among each other.

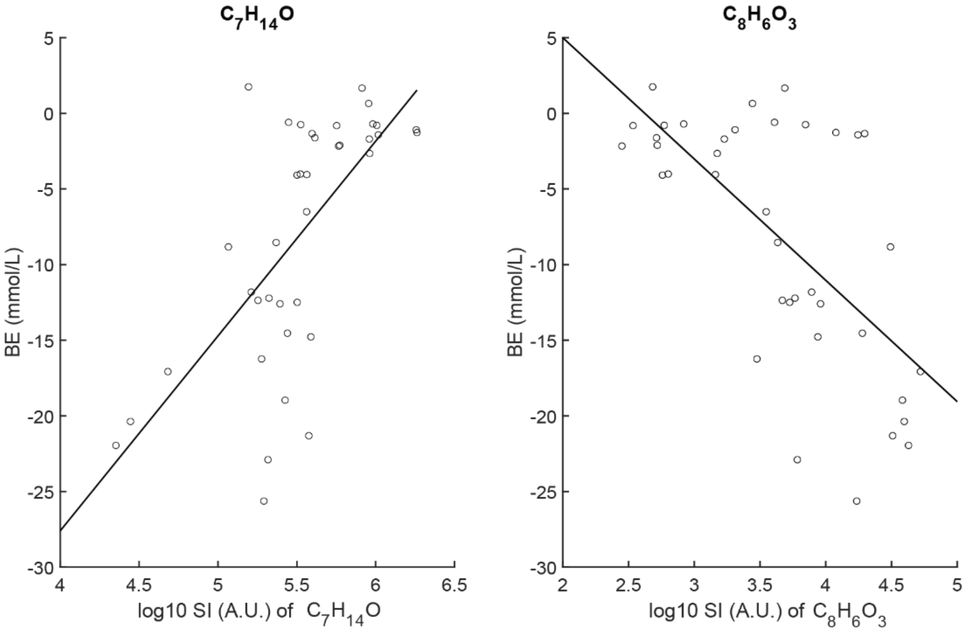

Figure S3 Two examples of features significantly correlating positively ($r = 0.67$; $q = 4x10^{-3}$) and negatively ($r = -0.66$; $q = 5x10^{-3}$) with BE (see complete series in Table S8).

Metabolic trajectories of all patients

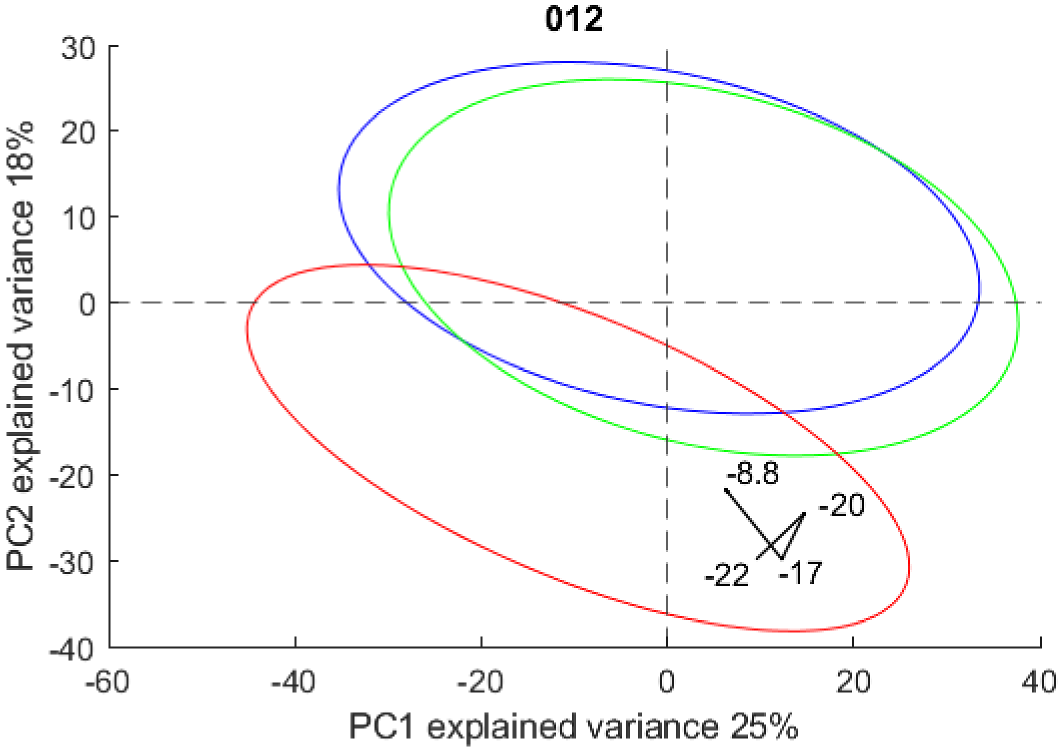

Figure S4 Trajectory of subject 012 during ICU stay (DKA). Subject 012, was the only DKA patient that displayed Kussmaul breathing. Its trajectory and BE values are well described in the BE<-2 section.

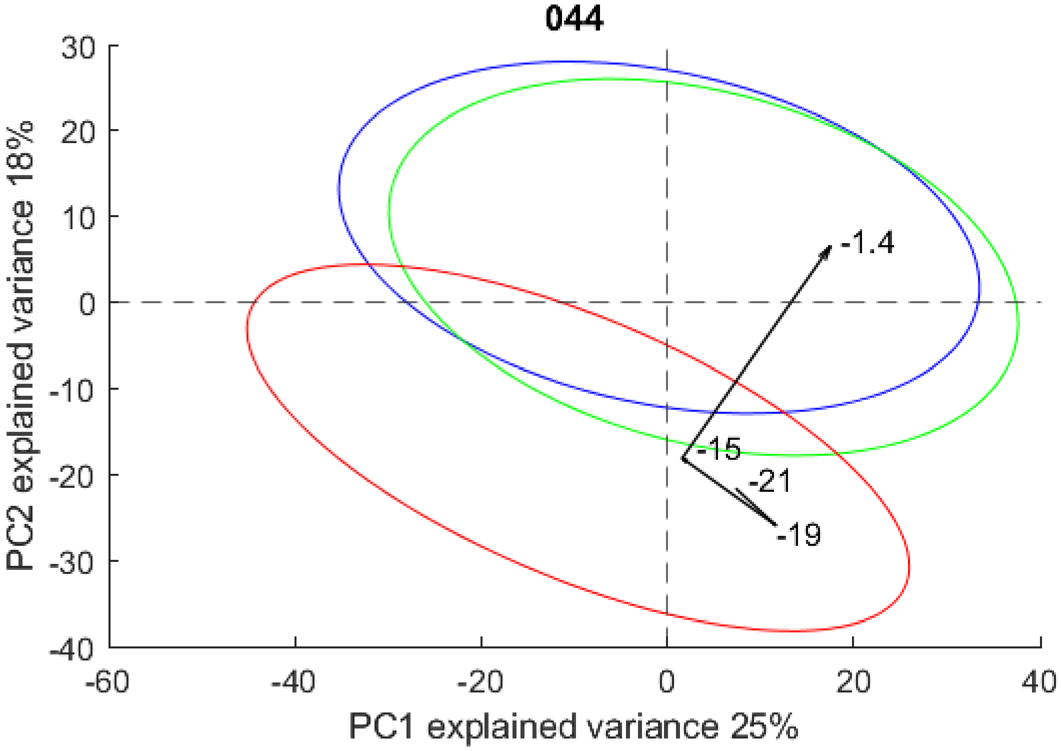

Figure S5 Trajectory of subject 044 during ICU stay (DKA). Subject 44 makes a clear trajectory towards in-control after scattering in the BE<-2 region.

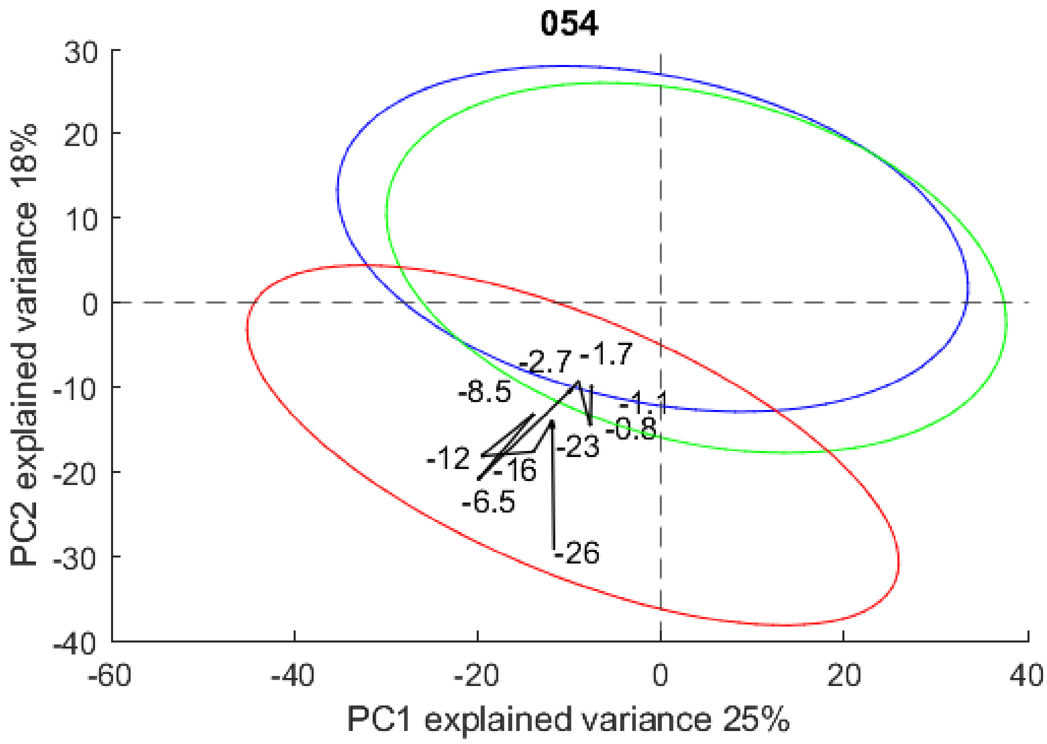

Figure S6 Trajectory of subject 054 during ICU stay (DKA). Subject 054 is a reoccurring DKA participant. Interestingly, the trajectory slowly drifts towards in-control and shows a less clear path towards in-control region.

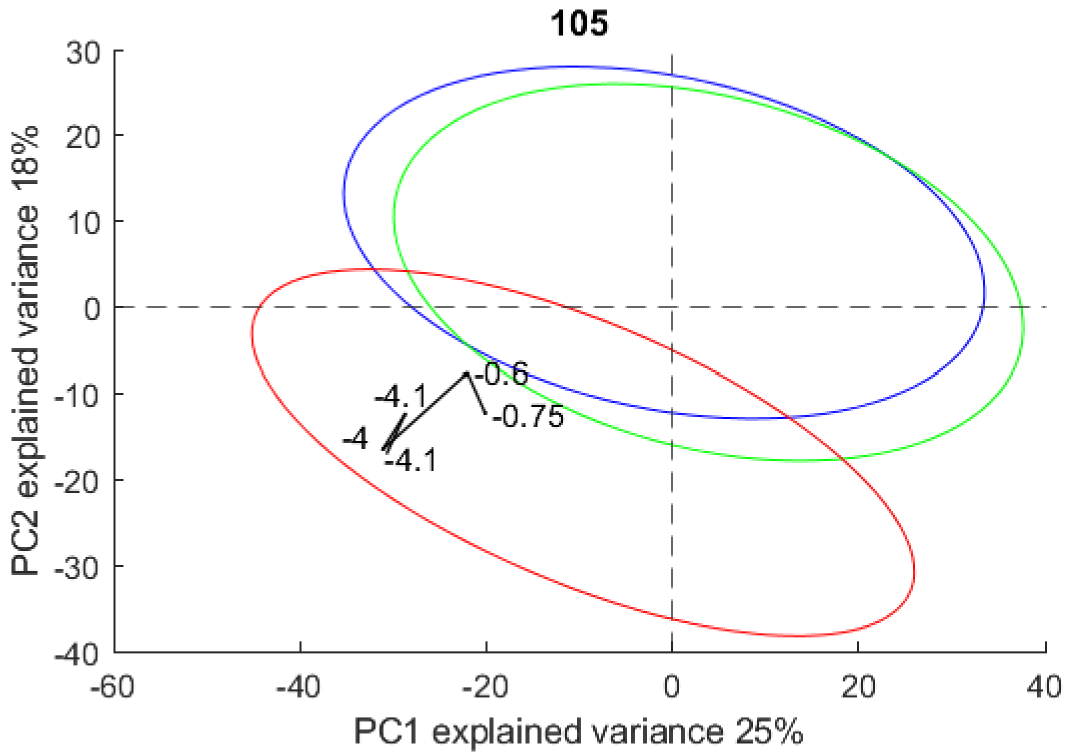

Figure S7 Trajectory of subject 105 during ICU stay (DKA). Subject 105 is a reoccurring DKA participant and shows a less sharp trajectory towards in-control after insulin therapy, similarly to subject 054.

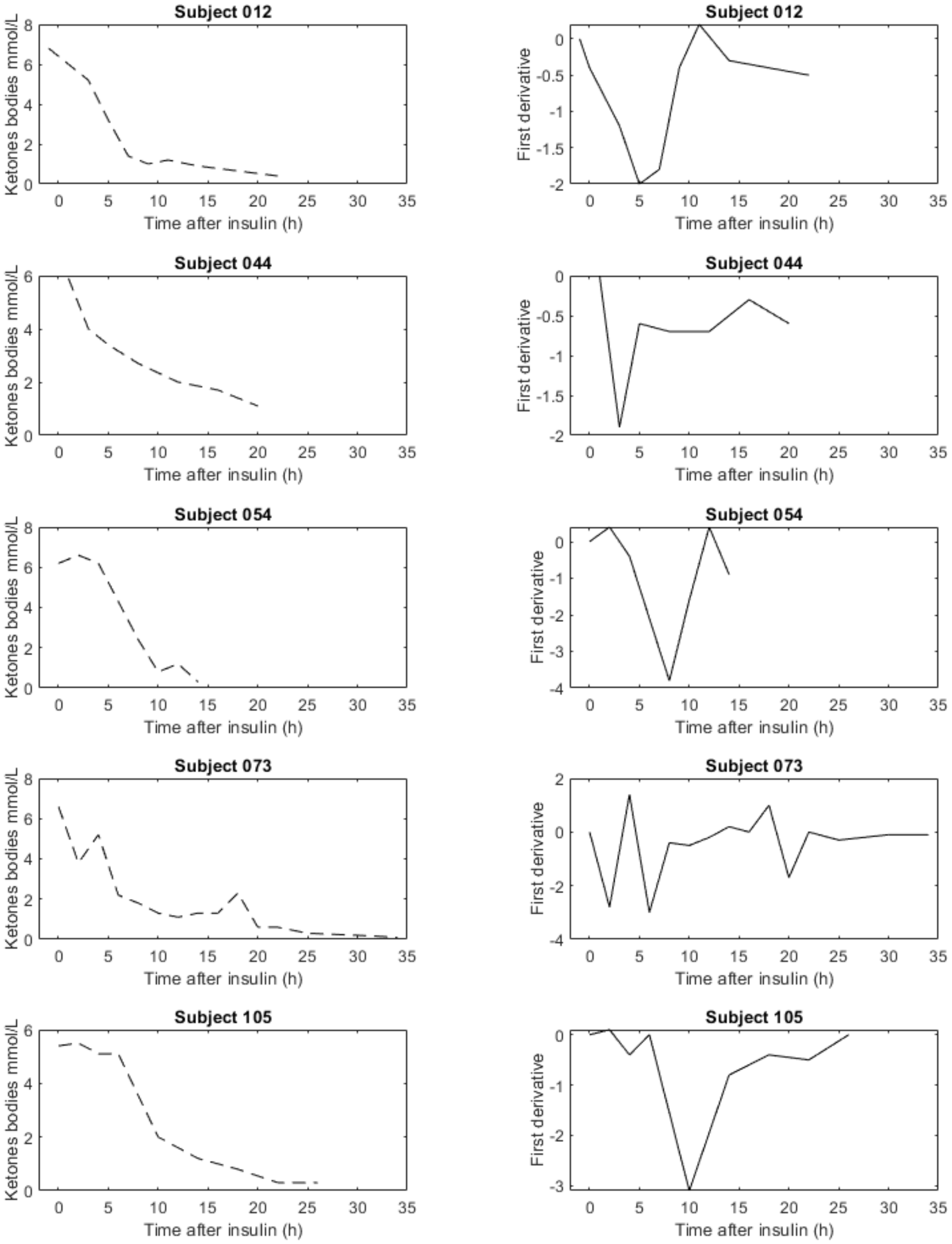

Figure S8 Time profiles of ketones (left) and its first derivative (right)

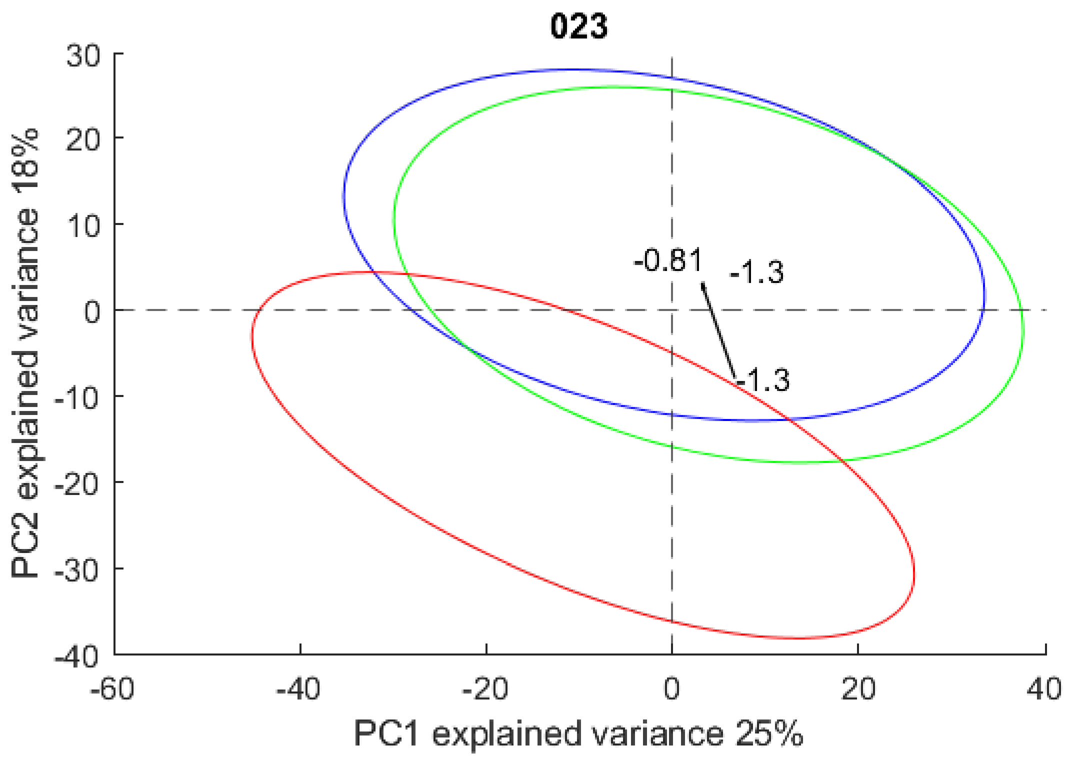

Figure S9 Trajectory of subject 023 during hospital stay (HG), its trajectory is well described in the in-control region which reflects its BE and clinical state.

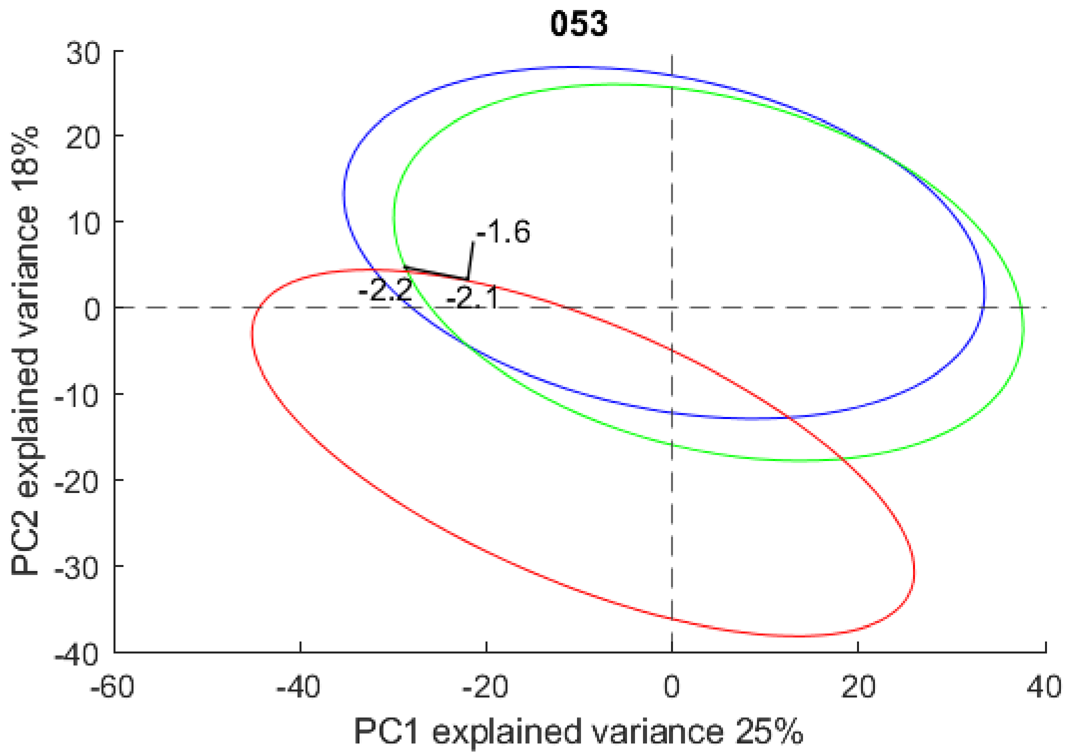

Figure S10 Trajectory of subject 053 during hospital stay (HG), its trajectory is well described in the in-control region however at the interface between BE<-2, which reflects its clinical state.
